# Patients with Type 2 Diabetes Mellitus failing on oral agents and starting once daily insulin regimen; a small randomized study investigating effects of adding vildagliptin

**DOI:** 10.1186/1756-0500-7-579

**Published:** 2014-08-29

**Authors:** Wendela Lucia de Ranitz-Greven, Joline Wilhelma Johanna Beulens, Lette Birgit Elisabeth Anne Hoeks, Gerdien Belle-van Meerkerk, Douwe Hedde Biesma, Harold Wessel de Valk

**Affiliations:** Department of Internal Medicine, University Medical Centre Utrecht, Huispostnummer, Postbus 85500 3508, Utrecht, GA F02-126 The Netherlands; Julius Centre for Health Sciences and Primary care, University Medical Centre Utrecht, Utrecht, The Netherlands

**Keywords:** Clinical trial, DPP4-inhibitor, Insulin, Therapy Vildagliptin

## Abstract

**Background:**

The addition of a DDP4-inhibitor to existing insulin therapy reduces HbA1c. However, no data exist about the addition of these agents at the beginning of insulin treatment in type 2 diabetes while this could especially be interesting because it is during this period that considerable residual beta cell function is still present. The benefit of such a strategy could be a lower insulin dose required for glycemic control. The hypothesis of our study was that adding a DPP4-inhibitor at the beginning of insulin treatment could lead to less exogenous insulin requirement, a reduction of hyperinsulinemia and side effects (hypoglycemia and weight gain), less glucose variability and improvement of insulin and glucagon dynamics during a mixed meal test.

**Results:**

In this small clinical trial (trial registration NTR2022) 9 patients were randomized to receive vildagliptin and 6 to receive placebo in addition to start of once daily insulin treatment. Unfortunately, due to a difficult inclusion, the preset sample size of 40 patients could not be met. Median units of insulin at the end of the study was 47 U in the placebo group and 34 U in the vildagliptin group. Median glycemic variability (SD) at the end of study was 2.1 in the placebo group and 1.5 in the vildagliptin group. Median weight gain at the end of study was 3 kg in the placebo and 0.5 kg in the vildagliptin group. Occurrence of hypoglycemia was low in both groups. Insulin, C-peptide, glucose and glucagon levels were comparable during mixed meal tests.

**Conclusions:**

This small randomized study did not have sufficient power to detect effects of the addition of vildagliptin to the start of once daily long-acting insulin. However in our opinion adding a DPP4-inhibitor, especially in this group remains a very interesting approach. This study could be used as a guidance for larger studies that are required to investigate the effects of this intervention on insulin requirements, glycemic variability, hypoglycemia and weight gain.

**Electronic supplementary material:**

The online version of this article (doi:10.1186/1756-0500-7-579) contains supplementary material, which is available to authorized users.

## Background

Due to the progressive nature of the disease, most patients with type 2 diabetes mellitus (DM) ultimately fail on oral glucose-lowering drugs and therefore require insulin therapy [1, 2]. Often considerable doses of insulin are needed and weight gain and hypoglycemia can occur [1]. The dipeptidyl peptidase-4 inhibitor (DPP4-inhibitor) vildagliptin is an oral glucose-lowering drug that leads to glucose-dependent insulin secretion and improvement of alpha cell function [3]. A reduction in glycated haemoglobin (HbA1c) and less hypoglycemia was shown in a previous study adding vildagliptin on top off an existing insulin regimen [4]. Multiple randomized-controlled trials investigating the effect of DPP4-inhibitor added to existing insulin have been performed since [4–14]; however, no data exist about vildagliptin use at the start of insulin treatment in patients with Type 2 DM. Adding a DPP4-inhibitor at the beginning of insulin treatment could especially be interesting because it is during this period that considerable residual beta cell function is still present. The benefit of such a strategy could be a lower insulin dose necessary for glycemic control. To study this, we titrated insulin in all patients to the best achievable degree of glycemic control. Therefore, we did not use HbA1c as the primary end-point, as most previous studies on DPP4-inhibitors and insulin did, but the required insulin dose instead.

The aim of the study was to investigate whether or not the addition of a DPP4-inhibitor to start of insulin treatment could lead to lower exogenous insulin requirements together with lower glucose variability, less weight gain, less hypoglycemia, and improved insulin and glucagon dynamics after a mixed meal test through improvement in alpha and beta cell function.

## Results

In this study 19 patients were screened and 4 excluded (reasons for exclusion in (Additional file 1). Nine patients were randomized to receive vildagliptin and 6 patients to placebo (Additional file 1). This study was designed to include 40 patients in 1 year. Since inclusion rate was much slower than expected, the inclusion period was extended by more than a year. However, the number of 40 patients could still not be met and inclusion was therefore terminated after 15 patients.

Table 1 shows the baseline characteristics, which were comparable in both treatment groups.

**Table 1:** **Base-line characteristics**

	Placebo	Vildagliptin
N	6	9
Age (years)	60 (39–67)	64 (42–67)
BMI (kg/m2)	32 (26–35)	32 (27–34)
Female (%)	17%	22%
Duration DM (years)	5.5 (1–23)	6.0 (2–15)
Prior medication		
metformin/SU/TZD/other (%)	100/67/0/33%	100/89/11/22%
Diabetic complications		
any microvascular complication (%)	17%	33%
any macrovascular complication (%)	0%	33%
BP (syst/diast in mmHg)	128/78	126/73
HbA1c at start of the trial (mmol/mol)	64 (53–74)	62 (57–73)
(%)	8.0 (7.0-8.9)	7.8 (7.4-8.8)
SD of glucose values (variability) at start of trial	1.8 (1.0 -2.7)	1.7 (1.2 – 2.6)

All values are in median (range) or percentages.

Any micro- or macrovascular complication was defined as the percentage of patients who had one or more complications as judged clinically by the investigator at the moment of inclusion.

Abbreviations: BMI = body mass index, DM = diabetes mellitus, SU = sulfonylurea, TZD = thiazolidinediones, BP = blood pressure, HbA1c = *glycated* haemoglobin, SD = standard deviation.

Results are summarized in Table 2. Median number of units of insulin in the placebo group was 47 and in the vildagliptin group 34. In both groups compliance was high and HbA1c decrease was comparable. Median glycemic variability (SD) in the placebo group was 1.8 at the beginning of the study and 2.1 at the end, in the vildagliptin group glycemic variability was 1.7 and 1.5 respectively. Median weight increase at the end of the study was 3 kg in the placebo and 0.5 kg in the vildagliptin group. Occurrence of hypoglycemia was low and comparable in both groups. Median systolic blood pressure change in the vildagliptin group was + 2.5 and −3 mmHg in the placebo group. LDL and SAF only showed very minor changes.

**Table 2:** **Results**

	Placebo	Vildagliptin
**Primary endpoint**		
Units insulin	47 (16 – 62)	34 (12 – 62)
**Secondary endpoints**		
Glycemic variability end of study (SD)	2.1 (1.1 – 2.8)	1.5 (1.0 – 3.6)
Change in weight (kg)	3 (−2.5 – 5.5)	0.5 (−2.6 – 4)
Hypoglycemia during the study (nr per pat)	1.5 (0 – 5)	1.0 (0 – 8)
Change in blood-pressure (mmHg)		
Systolic	−3 (−12 – 6)	2.5 (−14 – 10)
Diastolic	−1.5 (−9 – 3)	−0.5 (−6 – 11)
Change in LDL (mmol/L)	0 (−1 – 0.6)	−0.3 (−1.4 – 0)
Change in SAF (skin AGEs) (AU)	0.1 (−0.4 – 0.8)	0.15 (−0.4 – 0.6)
**Safety**		
Patients with one or more hypoglycemia (%)	67%	78%
Patients with severe hypoglycemia (%)	0%	0%
Patients with one or more AE (%)	100%	44%
Patients with one or more SAE (%)	0%	0%
**Other**		
Delta HbA1c (mmol/mol)	−6.5 (−18 - 7)	−6 (−25 - 4)
(%)	−0.6 (−1.6 – 0.6)	−0.5 (−2.3 - 0.3)
Compliance (% of tablets taken)	96%	98%

All values are in median (range) or percentages.

Compliance:% of tablets taken during the whole study period.

Delta HbA1c: HbA1c end-begin, −6 means a decrease of 6 points in HbA1c during the study, a number without – means an increase.

Because of the small sample size no p-values are shown.

Abbreviations; LDL = low-density lipoprotein, SAF = skin autofluorescence, AGEs = advanced glycation end products, AU = arbitrary units, AE = adverse event, SAE = serious adverse event, HbA1c = glycated hemoglobin.

Comparable levels of glucose, insulin, C-peptide and glucagon were observed in both groups after mixed meal tests (Additional file 2: Figure S2A-D). Median values of area under curve (AUC) of glucose and glucagon were 1704 (1439–2279) and 5100 (3030–7620) in the vildagliptin group and 1773 (1439–1931) and 5310 (3525–10785) in the placebo group. Median levels of AUC of insulin and C-peptide were 5295 (1695–24705) and 343050 (106395–963000) in the vildagliptin group and 3795 (1710–7095) and 199703 (134550–281250) in the placebo group. Adverse events were reported more often in the placebo group. These consisted of flu like symptoms (3 patients, all in the placebo group), tiredness (1 vildagliptin, 1 placebo), toothache/parodontitis (1 vildagliptin, 1 placebo), a cold (1 vildagliptin), headache and diarrhea (1vildagliptin), reversible increase in gGT (1 placebo, max 165 U/l (normal value: 0–40 U/L)), sensation of tingling in the left arm, which was reversible during the study (1 placebo), mild orthostatic symptoms (1 vildagliptin). None of these events were considered to be severe or related to the study medication.

## Discussion

This is the first study investigating the addition of a DPP4-inhibitor to the *start* of once-daily long-acting insulin in insulin-naïve patients. Although this study was set up as a double-blind parallel-arm placebo-controlled trial, the sample size is limited and therefore we cannot make conclusions about the effect of addition of vildagliptin on units of insulin at the end of the study, variability, weight, hypoglycemia or response of insulin, C-peptide or glucagon after a mixed meal test.

This important limitation is due to difficulties in patient recruitment. In the Netherlands, most patients starting with once-daily insulin are treated by a general practitioner and not in an (academic) hospital, where this study was performed. Despite extensive collaboration with many general practitioners and hospitals in the region, patients were still difficult to recruit. The small sample size is therefore too small to have statistical power to confirm or reject the null hypothesis.

However, given the working mechanism of DPP4-inihibitors and our small sample size, we think it would be very interesting to investigate our hypothesis in a larger study. Our small study could potentially serve as a guidance for such larger studies. From our data it can be derived that a repeat study would need approximately 46 patients per group. This is based on a median units of insulin of 40 (SD 17) and 10 units of insulin decrease, which would be clinically significant (and 25% is comparable to the difference in median units of insulin in our study of 28%). This sample size calculation must be considered as a rough estimation, given the small sample size and not-normal distribution of our main end-point, but at this moment the best estimation available.

Besides the smaller sample size our study differs in two major design aspects from previous randomized-controlled trials. First, previous trials investigated the effect of add-on DPP4-inhibition therapy to *existing* insulin regimen [4–14], whereas we investigated the addition of vildagliptin to the *start* of insulin therapy in our study. We hypothesized that adding a DPP4-inhibitor to start of insulin treatment could lead to less exogenous insulin requirements. This could lead to a reduction of hyperinsulinemia, which is thought to have atherogenic and mitogenic effects [15]. Moreover, lower insulin use could reduce side effects of insulin treatment such as hypoglycemia and weight gain [1]. The addition of a DPP4-inhibitor to an existing insulin treatment without the intention to change the insulin regimen as previous trials did does not reflect clinical practice in which physicians will choose to alter insulin schedules. Only in one randomized trial the insulin dose was changed as a goal in one arm (insulin-increasing arm) and compared to the addition of sitagliptin to existing insulin regimen. In that trial, a difference of 25% in insulin dose was described between the insulin-increasing and the insulin-sitagliptin group [11] together with a more pronounced HbA1c decrease in the sitagliptin group. The 25% decrease in units of insulin found in that study is comparable to the magnitude (28%) we found.

Second, we used a different end-point compared to previous trials. In randomized-controlled trials about the effect of the addition of DPP4-inhibitors to insulin thus far, HbA1c or glycemic control were used as primary end-point [4–14]. We chose required units of insulin since, in our study, we added vildagliptin to the start of insulin and we aimed at the best glycemic control with insulin glargine in all patients.

Because a lower insulin dose may lead to less side effects, we also investigated these as secondary end-points. The first was hypoglycemia. With improvement of glycemic control, the incidence of hypoglycemic episodes was expected to increase. This was indeed observed in a study using sitagliptin [14]. In contrast, a study by Fonseca et al. showed less hypoglycemia together with a decrease in HbA1c when vildagliptin was added to an insulin regimen [4], although this could not be confirmed in a study by Kothny et al., using the same DPP4-inhibitor [10]. Less hypoglycemia, as shown in the study by Fonseca, could be a result of an improved alpha cell function, which has been described not only postprandial (reduction in glucagon) but also during the reaction after induced hypoglycemia when a slightly increased increment of glucagon was seen [3]. In our study occurrence of hypoglycemia was low in both groups. The second secondary end-point studied was weight. In contrast to GLP1-analoga, DPP4-inhibitors do not show a large effect on weight [16]. Differences in body weight were not shown in previous randomized-controlled trials with DPP4 inhibitors in combination with insulin [4–14], except for one study which compared insulin-increasing therapy with addition of sitagliptin to existing insulin regimen [9]. The design of that study most resembled ours, and it also found a difference in required units of insulin between the groups. Furthermore, that study showed less weight increase in the DPP4 addition group (between-group difference 1.7 kg in favour of the DPP4-group, which is in concordance in magnitude with our findings (vildagliptin group (+0.5 kg), placebo group (+3 kg).

We also studied additional end-points, including glucose variability. We hypothesize that since variability is determined by hyperglycemic and hypoglycemic excursions, effects on alpha cell function could result in less glucose variability. A decrease in postprandial glucagon could reduce hyperglycemic excursions, while an increase in glucagon response following a hypoglycemia could reduce a hypoglycemic excursion. A decrease in variability has been reported before from single-arm trials, investigating the effect of addition of a DPP4-inhibitor to insulin [17, 18]. In our study, median glycemic variability (SD) at the end of the study was 2.1 in the placebo group and 1.5 in the vildagliptin group. We evaluated responses of insulin, C-peptide and glucagon during a mixed meal test, which were comparable in our study. It is difficult to assess insulin and glucagon dynamics in patients taking insulin [14]. All patients were on stable insulin dose, titrated at the fasting glucose levels and all patients were fasting. However, since exogenous insulin was present at time of testing because all patients took their glargine at bedtime the day before the testing, we cannot exclude that measurements are confounded by exogenous insulin. Since GLP1 could also have an effect on cardiovascular parameters, these were also included as an endpoint [19]. Levels for blood pressure, LDL and skin autofluorescence were comparable.

## Conclusions

This small randomized study did not have sufficient power to detect effects of the addition of vildagliptin to the beginning of once daily long-acting insulin. However in our opinion adding a DPP4-inhibitor, especially in this group remains a very interesting approach. This study could be used as a guidance for larger studies to investigate the effects of this intervention on insulin requirements, glycemic variability, hypoglycemia and weight gain.

## Methods

### Design

This study was set up as a double-blind parallel-arm placebo-controlled randomized monocenter trial (16 weeks) comparing the effects of adding vildagliptin or placebo to the start of insulin in patients with type 2 diabetes mellitus. Patients were included from December 2009 – May 2012.

### Patients

Patients who were scheduled by their treating physician to start once-daily long-acting insulin were eligible. Inclusion criteria were: Type 2 DM, failing on maximally tolerated oral-glucose-lowering medication, BMI 25–35, HbA1c 53–75 mmol/mol (7.0-9.0%) and age 25–75 years. Exclusion criteria were: pregnant women or women in the fertile period of life without adequate birth-control, type I DM, or another type of DM (for example pancreatic injury, prednisone induced), acute metabolic diabetic complications during the last 6 months, severe cardiac (left ventricle ejection fraction (LVEF) < 30%) or (a history of) hepatic failure (transaminases > 3 times elevated), or renal impairment (creatinine clearance <50 ml/min).

### Randomization

Patients were randomized to receive vildagliptin (50 mg twice daily) or matching placebo (twice daily). To prevent confounding by BMI, patients were randomized after stratification for BMI (using two different randomized lists for BMI 25–30 kg/m^2^ or BMI 30–35 kg/m^2^). Consecutive patients were allocated to the two different groups using two computer-generated randomized lists (block size 4), which were stored in sealed envelopes. Randomization and distribution of blinded study medication was performed by a person not related to the study (pharmacy). Patients and care providers were unaware of the randomization code.

### Treatment

Besides the study medication, all patients started with once daily long-acting insulin glargine at bedtime in combination with a fixed dose of metformin (twice daily 850 mg). Other glucose lowering drugs were terminated. The combination with metformin was used since this is standardized approach in Dutch clinical practice. The dose of insulin glargine was protocolized and titrated based on daily fasting blood glucose measurements of the patients and an algorithm comparable to the one published by Davies et al. [20]. In short, all patients started with 8 units insulin glargine at bedtime and performed daily fasting glucose measurements. The insulin dose was increased based on the mean of the last glucose measurements. This was performed twice a week for the first 3 weeks and once a week during the remainder of the study. The insulin dose was raised as follows: mean glucose 5.5-6.7 mmol/L: raise of 0–2 units, 6.7-7.8 mmol/L: 2 units, 7.8-10 mmol/L: 4 units, >10 mmol/L: 6–8 units increase. The dose was only raised in the absence of hypoglycemia.

### Outcomes

Primary outcome: units of insulin at the end of the study.

Secondary outcomes: 1) glycemic variability estimated by the standard deviation (SD) of 48 hours glucose values at the end of the study as measured by three days blinded continuous glucose measurement (CGM)) [21] 2) change in weight 3) hypoglycemia (defined as the total number of hypoglycemia during the study period (any glucose self-measurement < 4.0 mmol/L or symptoms which the patients recognises as hypoglycemia and clinically judged by the investigator as hypoglycemia)) 4) severe hypoglycemia (defined by help needed from others, seizure, or coma) 5) alpha and beta cell function (between group comparison of glucose, insulin, C-peptide and glucagon levels, measured after standardised meal) 6) cardiovascular analysis; change in blood pressure (mean ambulant 24 hour arterial pressure) and change in plasma lipids 7) change in skin Advanced glycation end products (AGEs) (measured as skin-autofluorescence by the AGE-reader) [22, 23].

Adverse events (AE): any (worsening of an) undesirable sign, symptom, or medical condition that was noted by the patient occurring after starting the study drug even if the event was not considered to be related to the study drug.

Serious adverse events (SAE): AE judged as medically significant, requiring in-patient hospitalization, prolongation of existing hospitalization, or resulting in persistent or significant disability or incapacity or are life-threatening or fatal.

### Study procedures

Age, BMI, duration of DM, medication use, history of diabetic complications, blood pressure, prior hypoglycemia (defined as symptomatic hypoglycemia in the past year), HbA1c, glucose variability and laboratory values were recorded at baseline. Patients were seen every 4 weeks in the out-patient clinic in our academic hospital and with telephone consultation every week. Drug accountability and laboratory measurements were performed at 8 and 16 weeks. CGM and ambulant blood pressure measurement were repeated at the end of the study. At the end of the study a standardised mixed meal test (MMT) was performed. Patients arrived fasting at the outpatient clinic and took their medication (metformin and study medication) at t = 0, after blood samples were drawn as a baseline (t = 0). At t = 30 blood samples were drawn again and they consumed a standardized breakfast within 15 minutes (containing 522 Kcal; 27 gr protein, 60 gr carbohydrates, 18 gr fat). Blood samples for glucose, insulin, C-peptide and glucagon were then drawn at t = 45, 60, 90, 120, 150 and 210 minutes. Insulin and C-peptide were measured using an electrochemiluminescence immunoassay on the Modular E170 (Roche Diagnostics GmbH, D-68298 Mannheim, Germany). Finally glucagon was measured with a competitive radioimmunoassay (Glucagon KGND1, Siemens Healthcare Diagnostics Inc, Los Angeles, USA).

The study was conducted using Good Clinical Practice according to the declaration of Helsinki. The protocol was approved by the ethics committee of the UMCUtrecht and all patients provided written informed consent. This investigator-driven study was designed, performed and analysed by the researchers of the UMCUtrecht. Novartis provided study medication and a research grant. The protocol of this trial was published before start of the trial at www.trialregister.nl (NTR2022).

### Sample size calculation

The group size was based on the primary study parameter: the absolute difference in daily insulin dose of long-acting insulin between the two groups at the end of the trial. A previous study in patients starting on once-daily insulin showed a mean insulin glargine dose of 57 +/− 15 units [24]. A meaningful decrease would be a difference of at least 14 units. A similar decrease (25%) in insulin dose has been observed when adding metformin to existing insulin treatment [25]. With a beta of 0.2 and a one-sided alpha of 0.05, the minimum number of patients in each group is 15. To correct for unforeseen circumstances, we planned to include 20 patients in each group.

### Statistics

Results are presented as median and range, categorical parameters as percentages. Areas under the curve of glucose, insulin, C-peptide and glucagon were calculated using the trapezoidal method. Since we did not meet our intended sample size results are presented in a pure descriptive way.

## Electronic supplementary material

Additional file 1: Figure S1: Randomization scheme of the trial. (GIF 14 KB)

Additional file 2: Figure S2: Glucose, insulin, C-peptide and glucagon levels after mixed meal test. (PDF 121 KB)

## Abbreviations

AE: Adverse event
AGEs: Advanced glycation end products
AUC: Area under curve
BMI: Body mass index
CGM: Continuous glucose measurement
DM: Diabetes mellitus
DPP4-inhibitor: Dipeptidyl peptidase-4 inhibitor
GLP1: Glucagon-like peptide-1
HbA1c: Glycated hemoglobin
LVEF: Left ventricle ejection fraction
MMT: Mixed meal test
SAE: Serious adverse event
SD: Standard deviation.
