# Supplementary figures and images for: Patients with Type 2 Diabetes Mellitus failing on oral agents and starting once daily insulin regimen; a small randomized study investigating effects of adding vildagliptin
Source: BMC Res Notes. 2014 Aug 29;7:579. doi: 10.1186/1756-0500-7-579 (PMC4161897; doi:10.1186/1756-0500-7-579)

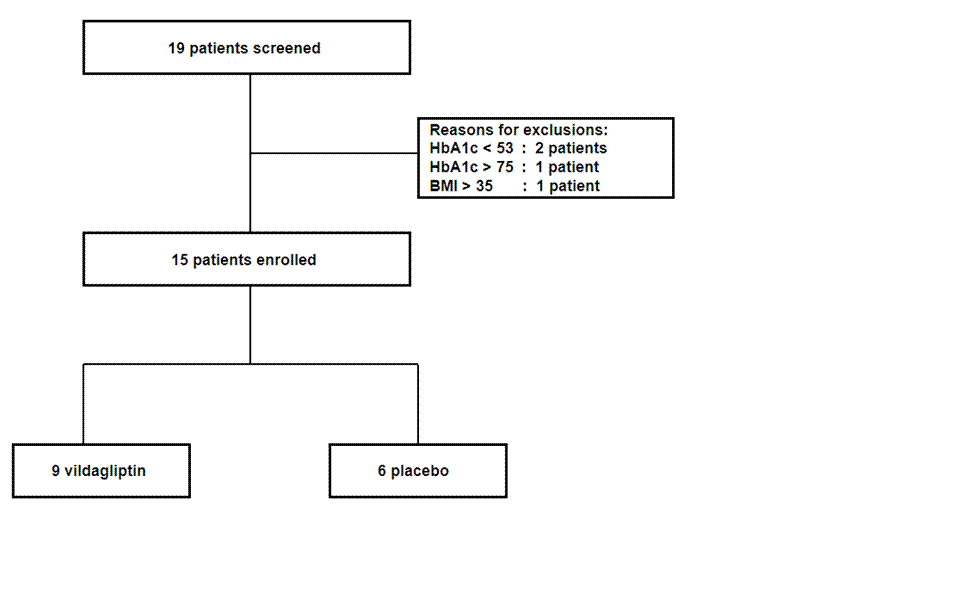

Supplement: Supplementary file 1 — Additional file 1: Figure S1: Randomization scheme of the trial. (GIF 14 KB) [file 13104_2014_3112_MOESM1_ESM.gif]

Glucose (mmol/L)

□ Placebo  
● Vildagliptin

Time (minutes)

↑ Study  
medication

↑ Meal

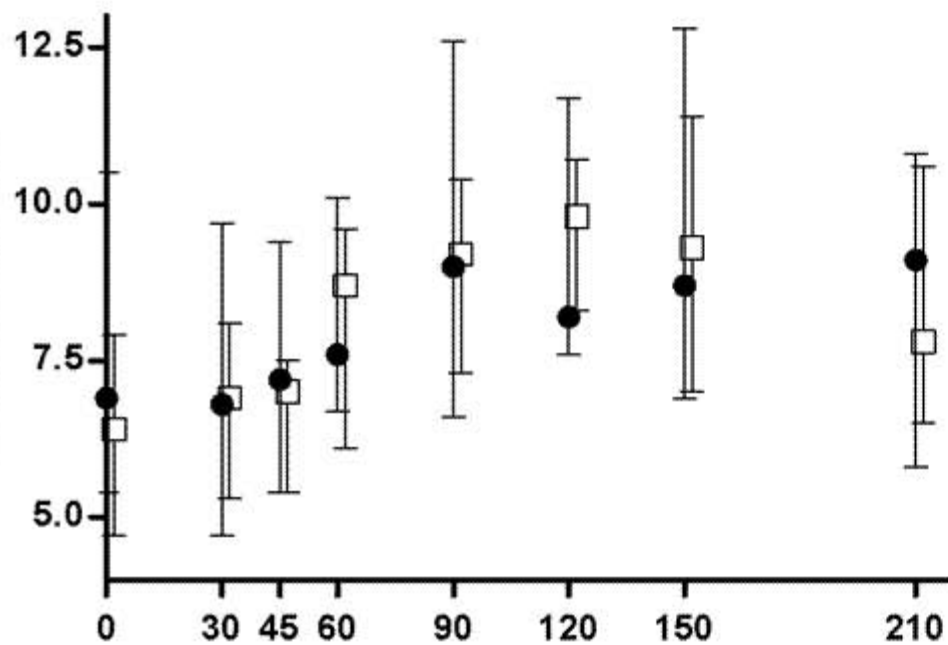

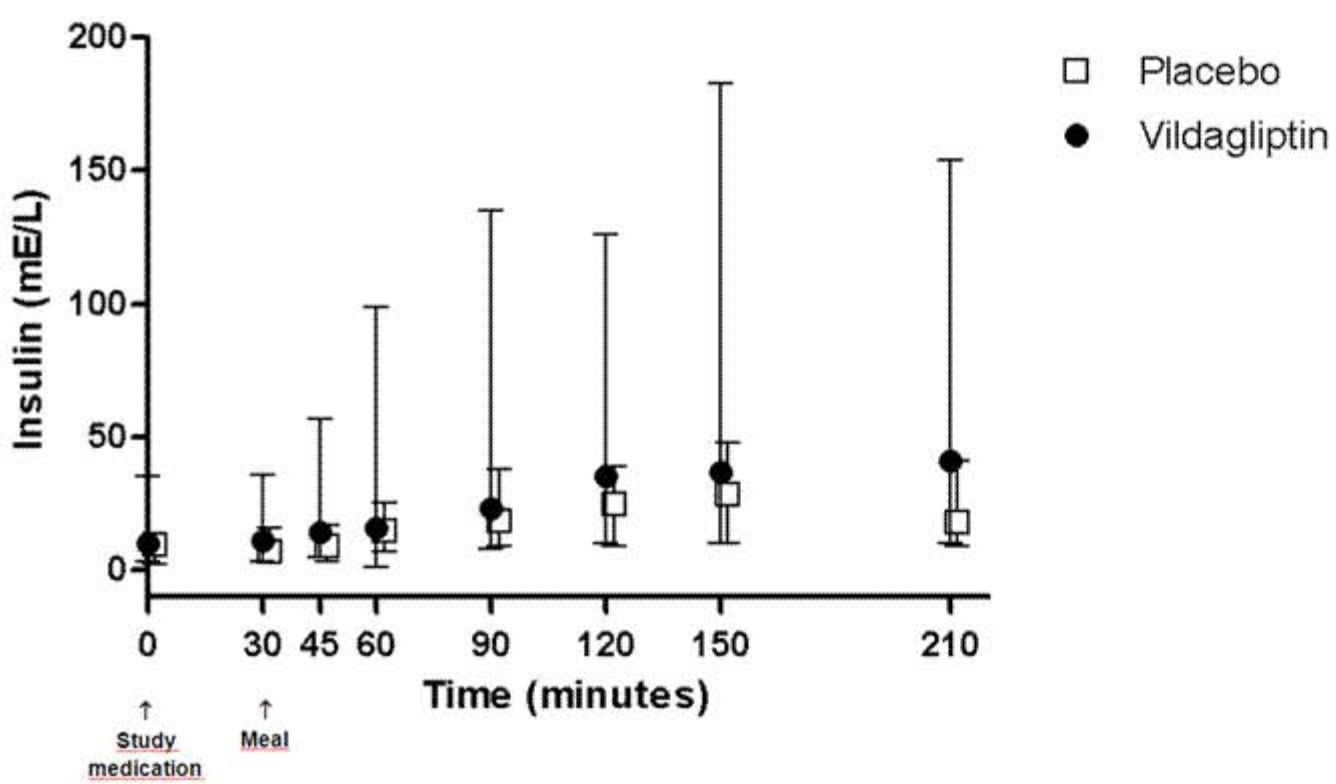

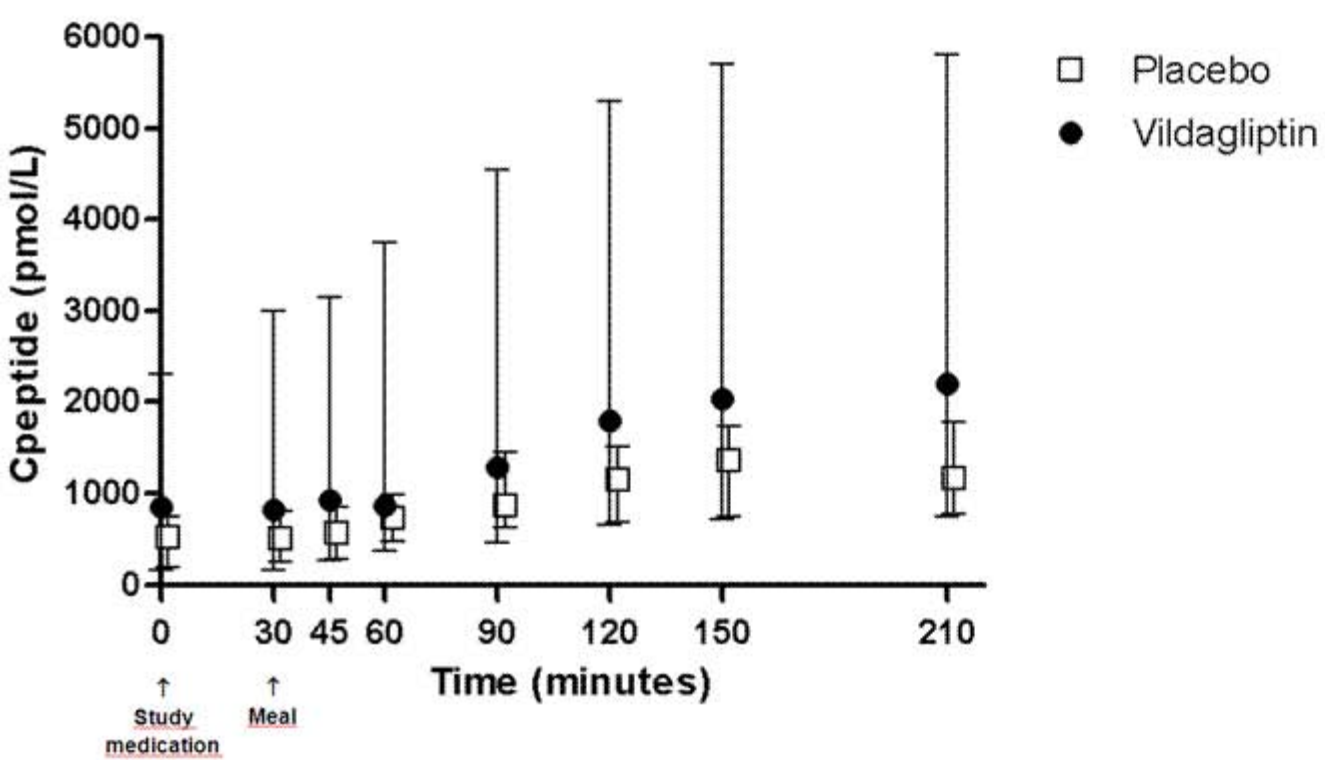

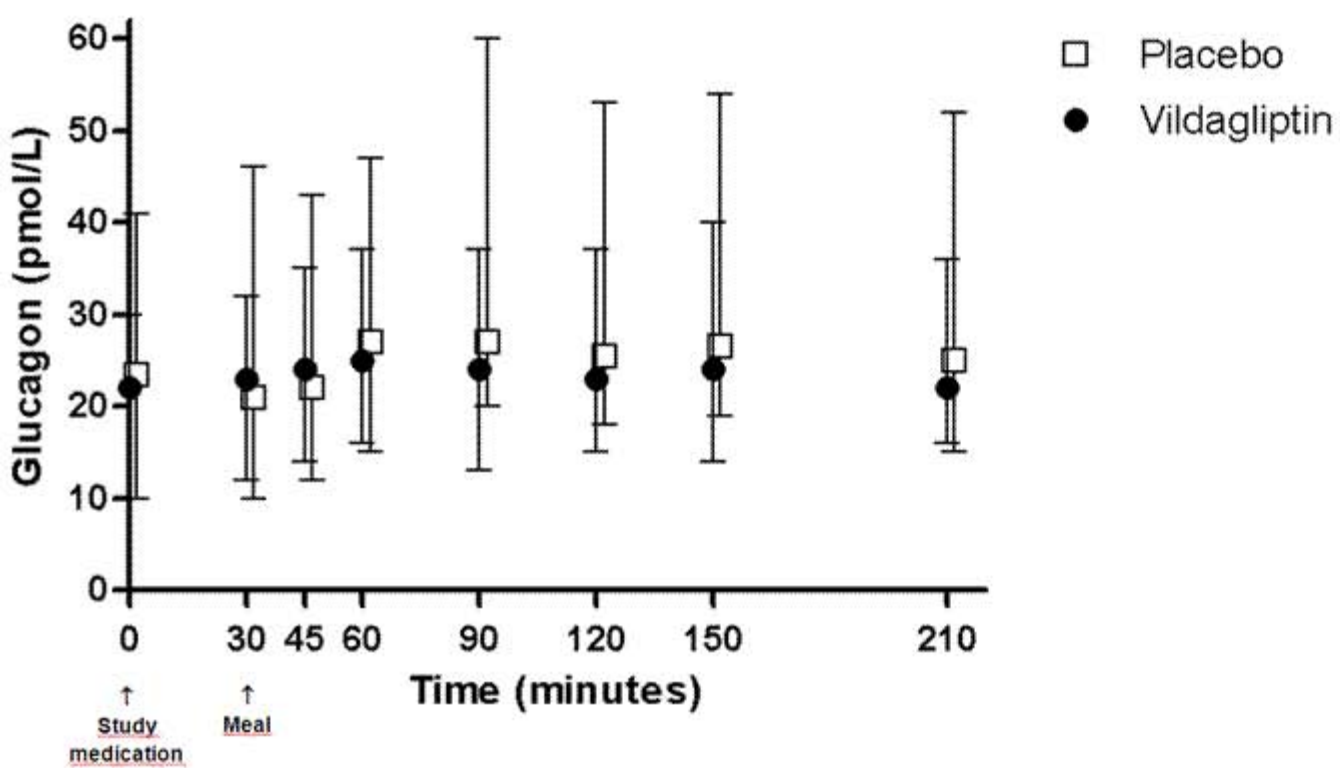

Supplement: Supplementary file 2 — Additional file 2: Figure S2: Glucose, insulin, C-peptide and glucagon levels after mixed meal test. (PDF 121 KB) [file 13104_2014_3112_MOESM2_ESM.pdf]
